# Chyle leak after transposition of the aberrant left vertebral artery via 1-debranching thoracic endovascular aortic repair: a case report

**DOI:** 10.1186/s44215-023-00033-6

**Published:** 2023-04-20

**Authors:** Kondo Nobuo, Wakisaka Hodaka, Kawashima Yuta, Kihara Kazuki, Noda Takahiro, Oue Kensuke

**Affiliations:** 1 Department of Cardiovascular Surgery, Kochi Health Science Center, Kochi, Japan; 2 Department of Radiology, Kochi Health Science Center, Ike 2125-1, Kochi city, Kochi, 781-8555 Japan

**Keywords:** Chyle leak, 1-debranched TEVAR, Vertebral artery transposition

## Abstract

**Background:**

Although the transposition of the aberrant left vertebral artery (ALVA) in 1-debranching thoracic endovascular aortic repair requiring zone 2 coverage for thoracic aneurysm with ALVA is reported to be an effective option, there are few reports of complications associated with the transposition of the ALVA.

**Case presentation:**

An 87-year-old man underwent 1-debranching thoracic endovascular repair for a saccular thoracic aortic aneurysm with the aberrant left vertebral artery. Simultaneously, the transposition of the ALVA was performed to prevent cerebral complications because the left vertebral artery was dominant. Postoperative computed tomography revealed the reconstructed vertebral artery and no endoleak. However, a postoperative chyle leak occurred and was treated with lymphangiography.

**Conclusion:**

Although the ALVA transposition is one of the better options, a chyle leak should be considered a serious complication of the procedure.

## Background

In thoracic endovascular aortic repair (TEVAR) requiring zone 2 coverage for thoracic aneurysm (TAA) with an aberrant left vertebral artery (ALVA), the transposition of the ALVA via 1-debranching TEVAR with a left common carotid artery-left subclavian artery (LCCA-LSCA) bypass is reported as an effective option for reducing the risk postoperative cerebral complications [1, 2]. However, there are few reports of complications associated with the transposition of the ALVA.

Herein, we report a case of chyle leakage (CL) after the transposition of the ALVA via 1-debranching TEVAR.

## Case presentation

**Fig. 1:**
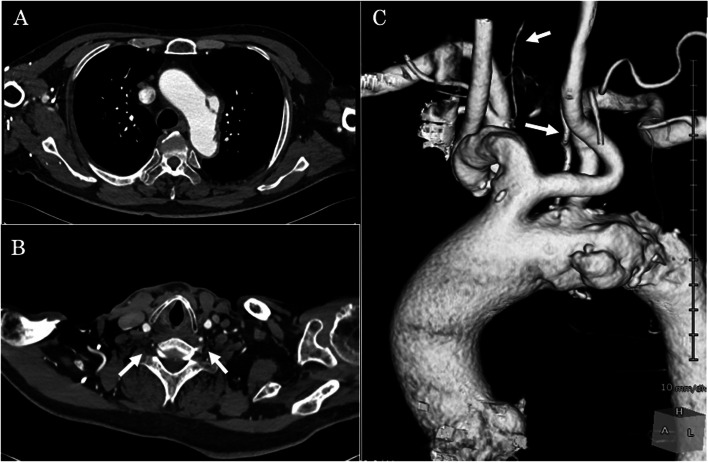
Preoperative computed tomography findings. **A** The CT revealed a bovine aortic arch and a saccular aortic aneurysm. **B** The arrows indicate vertebral arteries. The diameters of the left and right vertebral arteries (VA) were 4.5 mm and 1.7 mm, respectively. The right VA was hypoplastic. **C** The arrows indicate vertebral arteries. The aberrant left vertebral artery (ALVA) originated from the aortic arch between the bovine neck and the left subclavian artery. The ALVA ran along the back side of the left common carotid artery at the neck

An 87-year-old man with hypertension and chronic kidney disease (eGFR 42.9 mL/min/1.73 cm^2^) was referred to our hospital for surgery based on a saccular aneurysm involving the aortic arch. Enhanced computed tomography (CT) revealed a bovine aortic arch and a saccular aortic aneurysm with a vertical diameter of 34 mm and a horizontal diameter of 50 mm (Fig. 1 A, C). The ALVA was indicated to originate from the aortic arch between the bovine neck and the *LSCA* and ran along the back side of the LCCA at the neck between the anterior scalene and long coli muscles to the sixth foramen transversarium of the cervical vertebra. Additionally, enhanced CT revealed that the diameters of the ALVA and the right vertebral artery were 4.5 mm and 1.7 mm, respectively, and that the right vertebral artery (VA) was hypoplastic (Fig. 1 B, C). And the enhanced CT of the head indicated the connection of Willis’ arterial ring and no intracranial vascular stenosis or occlusion.

Although his clinical frailty scale was level 2, TEVAR was performed rather than conventional total arch replacement considering the patient’s age and a 13.1% operative mortality risk reported in the Japan Adult Cardiovascular Surgery Database. We planned 1-debranching TEVAR with an LCCA-LSCA bypass and a transposition of ALVA because the proximal 2-cm landing endograft covered both the LSCA and the ALVA.

**Fig. 2:**
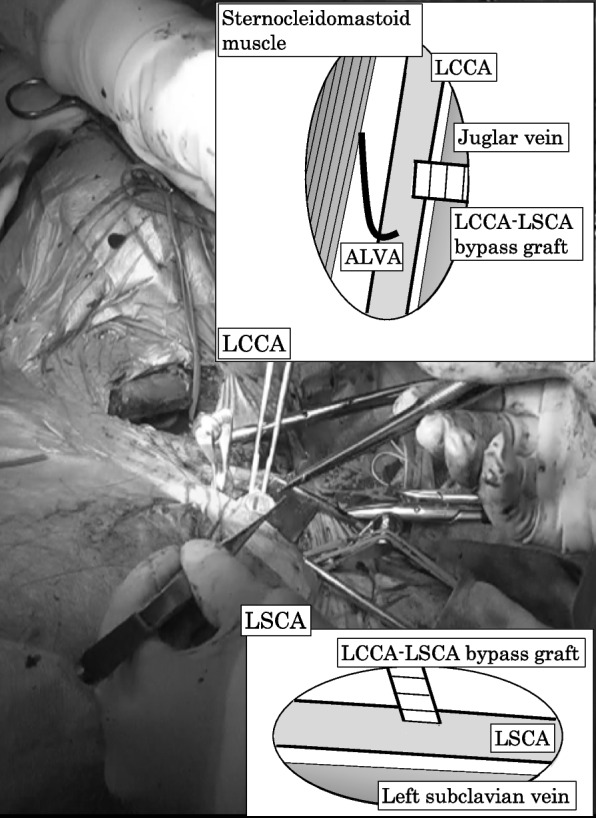
Intraoperative picture and illustrations. A 7-cm-skin incision was made along the posterior margin of the sternocleidomastoid muscle. The left common carotid artery (LCCA) was exposed and taped. Then, the aberrant left vertebral artery was easily exposed using the back side of the LCCA as a marker of the lower end of the thyroid cartilage. The left cubclavian artery was exposed at the other subclavian skin incision

The procedure was performed in a hybrid operating room under general anesthesia. The patient was positioned supine. The neck was advanced toward the right side. A 7-cm skin incision was made along the posterior margin of the sternocleidomastoid muscle. Firstly, LCCA was exposed and taped. Consequently, the ALVA was easily exposed using the back side of the LCCA as a marker of the lower end of the thyroid cartilage. The ALVA was easier to dissect because the ALVA moved freely caudally than at the level of the thyroid gland. On the cephalad side, the dissection of the ALVA was impossible because the ALVA ran through the intervertebral foramen. And we attempted to dissect the LSCA in the same field of view. However, we gave up to dissect the LSCA because the left subclavian vein and the left jugular vein were in the way. The LSCA was exposed at the other subclavian skin incision (Fig. 2). Systemic heparinization was intravenously taken prior to clamping the vessels. The LSCA-LCCA inner shunt was used because the peripheral LCCA pressure was 28 mmHg when the LCCA was clamped. The ALVA was ligated and transected before it was transposed to the LCCA using 7-0 polypropylene sutures in an end-to-side manner. The vertebral artery was then released. Subsequently, the LCCA was clamped at the distal position of this transposition. The 8-mm ringed vascular graft (FUSION vascular graft, Getinge, Göteborg, Konungariket Sverige) was anastomosed to the LCCA in an end-to-side manner and the vascular graft was anastomosed sequentially to the LSCA in an end-to-side manner, using 5-0 polypropylene sutures. The central side of the LSCA was untouched because the stent graft was thought to prevent retrograde blood flow.

The endovascular procedure immediately followed the ALVA transposition and LCCA-LSCA bypass. Conformable Gore TAG stent grafts measuring 45 mm × 15 cm and 31 mm × 15 cm (W.L. Gore & Associates, Newark, NJ, USA) were deployed in a retrograde fashion from the left femoral artery. Lastly, intraoperative angiography indicated patency of the debranching bypass graft and absence of endoleaks.

**Fig. 3:**
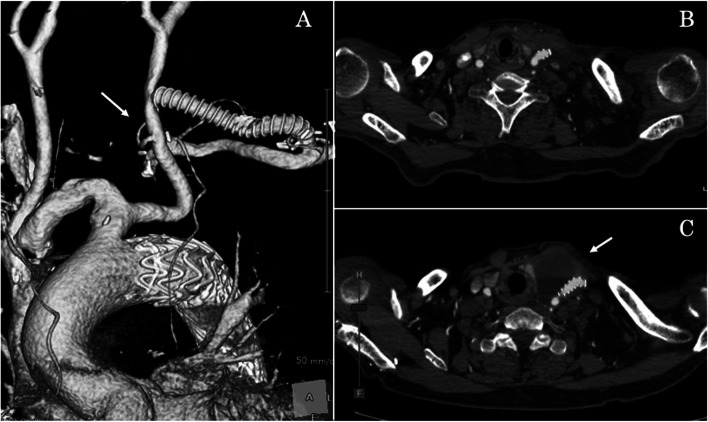
Postoperative computed tomography findings. **A**/**B** The arch aneurysm is excluded. The left subclavian artery-left common carotid artery bypass and the left vertebral artery (arrow) are patent. **C** The arrow indicates neck swelling caused by a chyle leak. **D** The picture shows puncture fluid of neck swelling caused by a chyle leak

The patient was extubated immediately after surgery. Despite having a transient cerebral ischemic attack with mild right hemiplegia, he was discharged without aftereffects on postoperative day 19. Similar to intraoperative angiography, postoperative CT revealed aneurysm exclusion, a patent debranching bypass graft, and no CL (Fig. 3 A, B).

On postoperative day 34, however, he was re-hospitalized for neck swelling caused by CL (Fig. 2 C, D). Notably, the trachea was displaced and compressed by the storage of CL. Upon puncturing the swelling area, approximately 150 mL of chyle was collected (Fig. 3). And the triglycerides were at 370 mg/dL. Firstly, he was conservatively treated with a fat-restricted diet and drainage of CL. And the drainage volume was 150–200 ml/day.

**Fig. 4:**
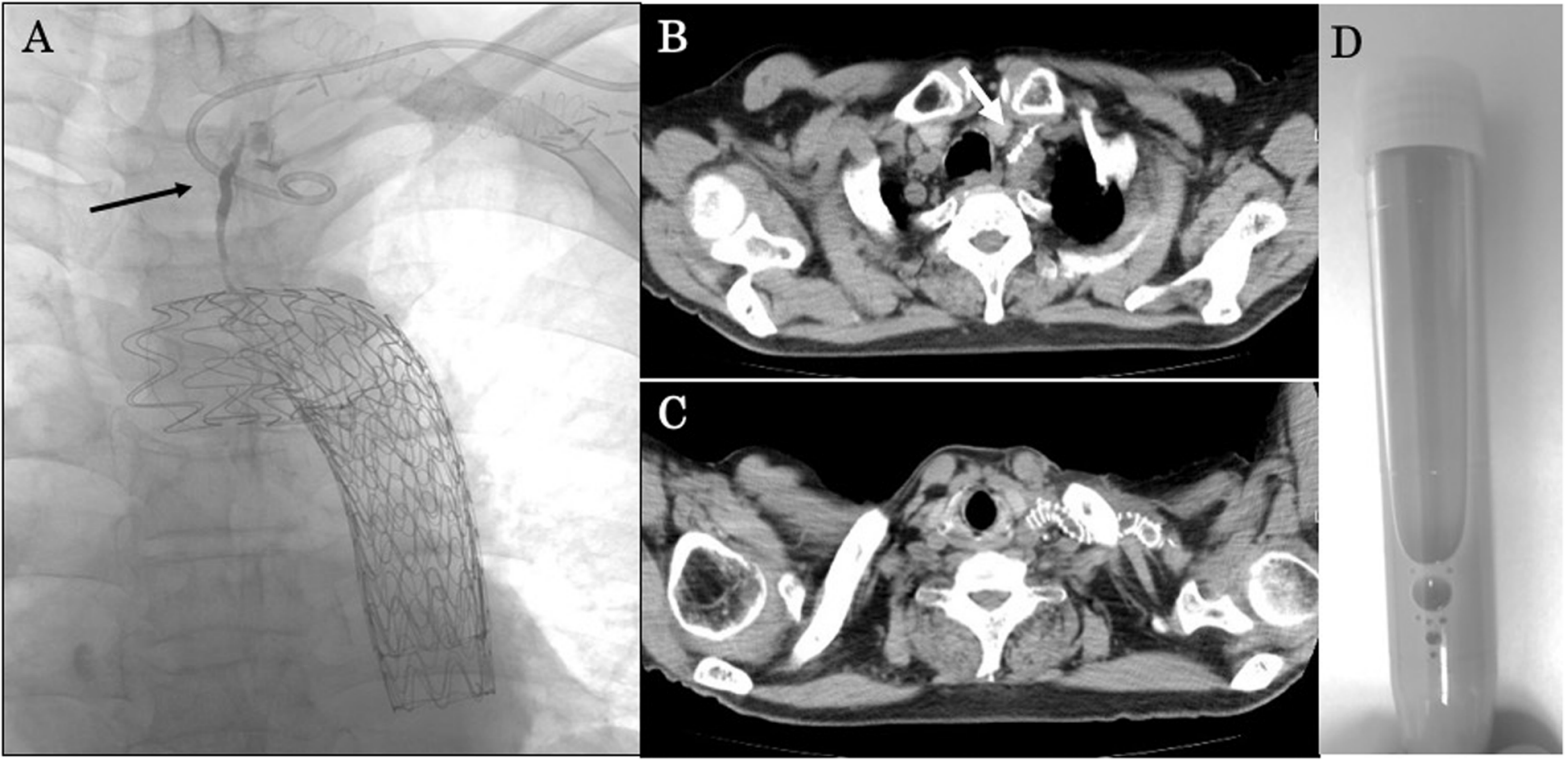
Lymphangiography and computed tomography after treatment. **A** Lymphangiographic radiography shows a drainage tube, abnormal lipiodol pooling in the chyle leak lumen, and obstruction of the point of leakage of the lymphatic vessel (the arrow). **B** Computed tomography after treatment revealed obstruction of the point of leakage of the lymphatic vessel (the arrow). **C** Computed tomography 3 months after discharge shows no reswelling on the neck

However, his neck reswelled. At the 9th day after the start of treatment, lymphangiography was performed with lipiodol (8.2 mL) to obstruct the lymphatic vessels from the left femoral lymph node (Fig. 4 A, B). The lymphangiography was successful, and CL decreased before gradually disappearing. CT revealed lipiodol retention inside the lymphatic vessel on the distal side of the point of leakage. As a result, he was discharged home without restorage of chyle. Restorage has not been observed 3 months after discharge (Fig. 4 C). Informed consent was obtained from the patient for publication of this case report and the accompanying images. And this case study has been approved by the ethical committee in our hospital (Clinical trial registration number: 221037).

## Discussion

The prevalence of an aberrant left vertebral artery (ALVA) is reported to be 0.8–6.6% according to some reports [3–5]. The ALVA typically originates from the aortic arch between the LCCA and the LSCA [3–5].

In TEVAR requiring zone 2 coverage, it is necessary to cover the left VA and LSCA to enable an adequate landing zone. While coverage of the left VA and LSCA is reported to be safe, Bradshaw et al. reported that the reduction of the flow of the left vertebral artery without revascularizing the LSCA and VA may increase the risk of postoperative cerebral complications [6, 7]. In this case, the reconstruction of ALVA was necessary because the ALVA was dominant and not hypoplastic. A previous study reported that the ALVA is easily transposed directly onto the left carotid artery^1^. Accordingly, we similarly performed transposition of the ALVA.

However, postoperative CL occurred in this case. CL caused by iatrogenic thoracic duct injury is a rare but serious and intractable complication in neck surgery with a rate of 0.5–8% [8, 9]. Moreover, most CL transpires with surgery of the left neck [8, 9]. In this case, we attempted to dissect the LSCA in the same field of view after the dissection of LCCA and ALVA was dissected. However, we gave up the dissection of the LSCA because the left internal jugular vein and the left subclavian vein were in the way. We thought that we unknowingly injured part of the lymphatic vessels because we dissected around the left venous angle at this time. And larger neck dissection in 1-debranching TEVAR with transposition of ALVA than without transposition was considered to be the cause of CL. It is difficult to confirm clearly the thoracic duct intraoperatively. The area around the venous angle is very difficult to visualize due to the structure of the skeleton. Therefore, it is important not to dissect the area around venous angle to prevent CL.

CL can be life-threatening if uncontrolled because of significant loss of fluid, plasma protein, fats, and immunoregulatory lymphocytes. While mortality is high in patients with uncontrolled or untreated CL, conservative and surgical interventions can successfully treat CL [8–10]. Conservative treatment involves a low-fat medium-chain triglyceride diet, total parenteral nutrition, drainage of CL, somatostatin treatment, and percutaneous embolism [8–10]. Surgical intervention can be considered if CL cannot be controlled with these conservative treatments. Notably, lymphangiography has been recently used as an effective therapeutic tool for CL. The mechanism of attenuation of CL by lymphangiography was considered to be as follows: [1] lipiodol infused in lymphangiography accumulated at the point of leakage outside of the injured lymphatic vessels, [2] a regional inflammatory reaction occurred in the soft tissue around the lipiodol leakage point, and [3] obstruction of the point of leakage of the lymphatic vessel ^10^. Based on this theory and our results, lymphangiography with lipiodol is an effective treatment for CL, although it was difficult to cure CL by diet and drainage alone.

## Conclusion

The transposition of the ALVA via 1-debranching TEVAR is an effective treatment option because of its relatively easy exposure. However, special care should be taken to prevent damage to the left thoracic duct to avoid serious iatrogenic complications like CL. To that end, lymphangiography with lipiodol could be an effective therapeutic tool for CL.

## Data Availability

Not applicable.

## Abbreviations

ALVA: Left aberrant left vertebral artery
TEVAR: Thoracic endovascular aortic repair
TAA: Thoracic aneurysm
LCCA: Left common carotid artery
LSCA: Left subclavian artery
CL: Chyle leak
CT: computed tomography.
